# Supplementary material for: A cell type enrichment analysis tool for brain DNA methylation data (CEAM)
Source: Epigenetics. 2025 Dec 22;21(1):2604360. doi: 10.1080/15592294.2025.2604360 (PMC12724277; doi:10.1080/15592294.2025.2604360)
Supplement: Supplemental Material [file KEPI_A_2604360_SM6672.docx]

The supplementary Tables and Figures are available on figshare repository ([https://doi.org/10.6084/m9.figshare.29986714.v4](https://doi.org/10.6084/m9.figshare.29986714.v3)).
